# Alginate hydrogel cross-linked by Ca^2+^ to promote spinal cord neural stem/progenitor cell differentiation and functional recovery after a spinal cord injuryhh

**DOI:** 10.1093/rb/rbac057

**Published:** 2022-08-18

**Authors:** Jun Zhou, Yaqi Wu, Zhijian Tang, Kaipeng Zou, Juan Chen, Zuowei Lei, Xueyan Wan, Yanchao Liu, Huaqiu Zhang, Yu Wang, Armin Blesch, Ting Lei, Shengwen Liu

**Affiliations:** Department of Neurosurgery, Tongji Hospital, Tongji Medical College, Huazhong University of Science and Technology, Wuhan 430030, China; Department of Neurosurgery, Tongji Hospital, Tongji Medical College, Huazhong University of Science and Technology, Wuhan 430030, China; Department of Neurosurgery, Tongji Hospital, Tongji Medical College, Huazhong University of Science and Technology, Wuhan 430030, China; Department of Anus-intestines, Chongqing University Affiliated Jiangjin Hospital (Jiangjin Central Hospital), Chongqing 402260, China; Department of Neurosurgery, Tongji Hospital, Tongji Medical College, Huazhong University of Science and Technology, Wuhan 430030, China; Department of Orthopedics, Tongji Hospital, Tongji Medical College, Huazhong University of Science and Technology, Wuhan 430030, China; Department of Neurosurgery, Tongji Hospital, Tongji Medical College, Huazhong University of Science and Technology, Wuhan 430030, China; Department of Neurosurgery, Tongji Hospital, Tongji Medical College, Huazhong University of Science and Technology, Wuhan 430030, China; Department of Neurosurgery, Tongji Hospital, Tongji Medical College, Huazhong University of Science and Technology, Wuhan 430030, China; Department of Neurosurgery, Tongji Hospital, Tongji Medical College, Huazhong University of Science and Technology, Wuhan 430030, China; Department of Neurosciences, University of California San Diego, La Jolla, CA 92093-0626, USA; Veterans Affairs San Diego Healthcare System, La Jolla, CA 92093-0626, USA; Department of Neurosurgery, Tongji Hospital, Tongji Medical College, Huazhong University of Science and Technology, Wuhan 430030, China; Department of Neurosurgery, Tongji Hospital, Tongji Medical College, Huazhong University of Science and Technology, Wuhan 430030, China

**Keywords:** regeneration, biomaterial, neural stem/progenitor cells, neural repair, spinal cord injury

## Abstract

Alginate capillary hydrogels seeded with differentiated cells can fill the lesion cavity and promote axonal regeneration after grafting into the injured spinal cord. Neural stem/progenitor cells (NSPCs) can potentially repair the spinal cord; however, effects of alginate hydrogels (AHs) on NSPCs remain unknown. In this study, we fabricated AHs cross-linked by Ca^2+^ and seeded hydrogels with rat embryonic day 14 NSPCs. Immunocytochemistry and electron microscopy show that NSPCs survive, proliferate and differentiate into neurons *in vitro* within the capillaries. After transplantation into an acute T8 complete spinal cord transection site in adult rats, approximately one-third (38.3%) of grafted cells survive and differentiate into neurons (40.7%), astrocytes (26.6%) and oligodendrocytes (28.4%) at 8 weeks post-grafting. NSPCs promote the growth of host axons within the capillaries in a time-dependent manner. Host axons make synapse-like contacts with NSPC-derived neurons within the hydrogel channels, and graft-derived axons extend into the host white and gray matter making putative synapses. This is paralleled by improved electrophysiological conductivity across the lesion and partial hindlimb locomotor recovery.

## Graphical Abstract

**Figure:**
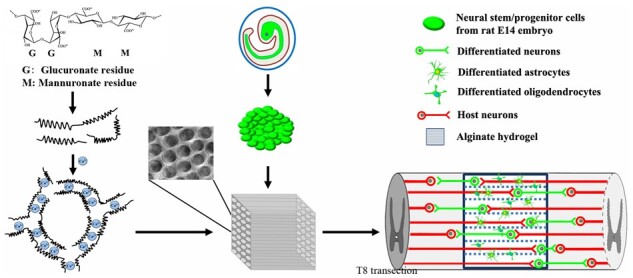

## Introduction

Spinal cord injury (SCI) caused by traumatic and nontraumatic incidents can result in a permanent locomotor deficits, sensory impairment and autonomic dysfunction [1]. After SCI, a complex cascade of inflammatory and pathological processes is triggered, resulting in neural death, cystic cavities and glial scar formation [2]. Endogenous stem cells do not restore the loss of adult neurons and glia in the injured area [3] limiting spontaneous tissue repair and functional recovery. Biomaterials in combination with cell transplantation is one potential means to effectively fill a lesion cavity, replace lost cells, facilitate axonal regeneration and attenuate sensorimotor dysfunction [4].

Among different biomaterials used in conjunction with cell transplantation, materials with high porosity and an anisotropic structure with linear channels can not only provide a substrate for co-transplanted cells and protect cells from a hostile inflammatory environment, but can also guide axons in rostro-caudal direction [5–7]. Alginates can be easily cross-linked to generate anisotropic capillary structures by diffusion of divalent cations from the top to the bottom of alginate solutions forming a stable hydrogel scaffold [8, 9]. Due to their excellent biocompatibility and low immunogenicity within the central nervous system (CNS) [8, 10], alginate hydrogels (AHs) have been widely investigated in neural tissue engineering as carriers for cellular grafts and biofunctional molecules [8, 9, 11]. The capillary diameter of AH ranging from 9 to 94 µm is dependent on the cations used: Zn^2+^ > Sr^2+^ > Cu^2+^ > Ba^2+^ [12]. AHs with anisotropic capillaries promote survival of grafted cells pre-loaded within the capillaries such as bone marrow stromal cells (BMSCs), Schwann cells (SCs) and neonatal astrocytes, which in turn enhance axonal growth [6, 13, 14]. Capillaries guide axonal growth in a linear pattern to increase axon growth into and across the lesion to reach the distal host parenchyma [7]. Previous studies with AH used differentiated cells to fill capillary structures prior to grafting. Effects of AH filled with neural stem/progenitor cells (NSPCs) in repairing the injured spinal have not been explored in detail.

Compared to differentiated cells, embryonic and induced pluripotent cell-derived NSPCs have several advantages for CNS diseases such as stroke [15, 16], traumatic brain injury [17] and neurodegeneration [18]. Multipotent neural stem cells isolated from the embryonic neural tube can generate neuron- and glial-restricted precursors, which can differentiate into mature neurons and glia, respectively [19, 20]. Caudalized NSPCs or NSPCs isolated from fetal spinal cord grafted into adult SCI lesions closely recapitulate neural development *in vivo* [21–23]. Corticospinal axons can regenerate into spinal cord NSPC grafts but not into neural progenitors with a rostral (brain) fate, which highlights the advantage of spinal cord NSPCs for SCI repair [24].

NSPCs cultured *in vitro* can be expanded as neurospheres with sphere size increasing over time. Although NSPC proliferation *in vivo* in a CNS lesion might be different from *in vitro* data, proliferation markers such as Ki-67 colocalize with nestin, a progenitor marker, in grafted cells even 3 months post-transplantation [25]. To allow for sufficient space for NSPC seeding and potential proliferation in AH capillaries and to arrange grafted cells in a linear pattern as axons in the normal white matter, AHs were fabricated using Ca^2+^ as cross-linking cation in this study. The resulting AH scaffolds displayed a larger capillary diameter than those cross-linked by other cations used in our previous studies [7]. Implantation of AHs loaded with rat embryonic NSPCs and grafted into a complete spinal cord transection fully bridged the gap and facilitated host axonal growth. NSPCs differentiated into neurons, astrocytes and oligodendrocytes and hindlimb locomotor function was significantly improved 8 weeks post-grafting.

## Materials and methods

### Fabrication of AH scaffolds with anisotropic capillaries cross-linked by Ca^2+^

**Figure 1.**
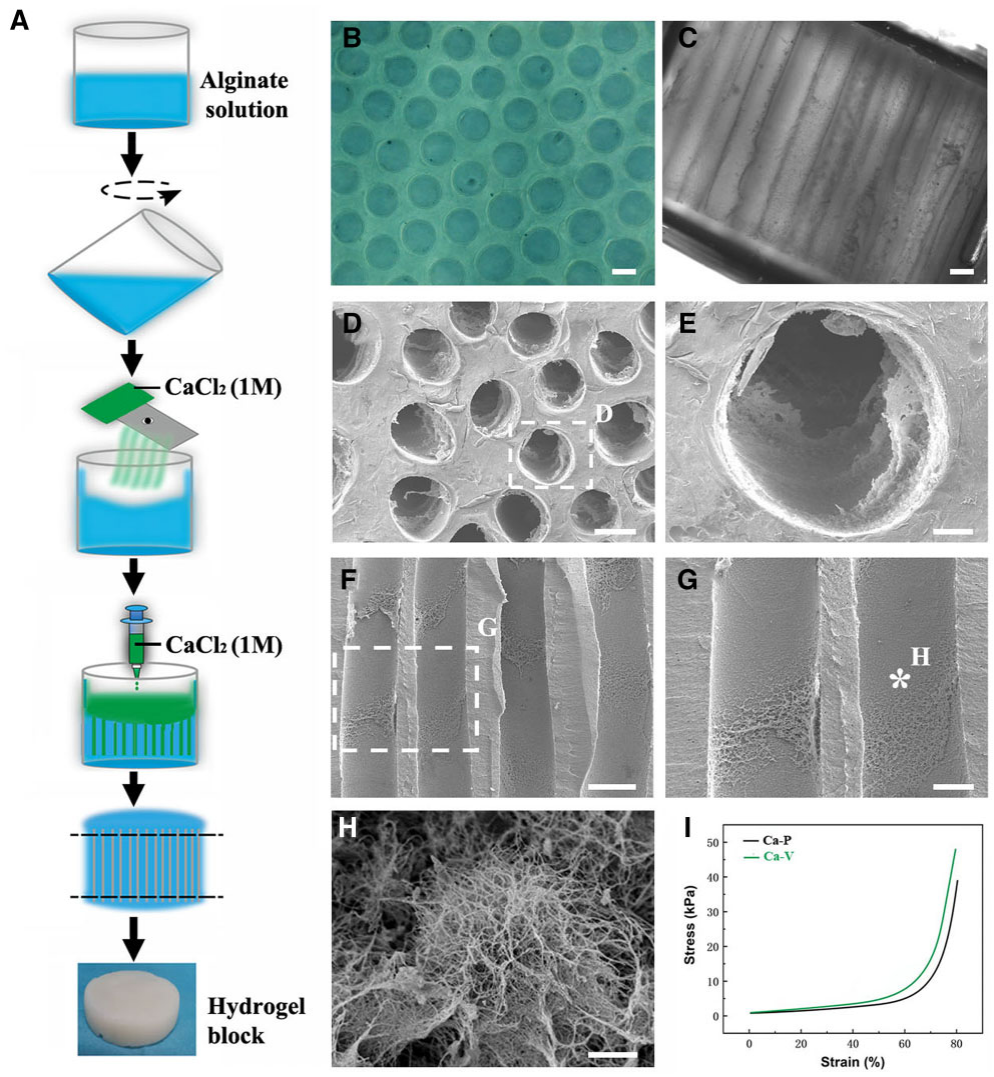
Fabrication and characterization of AHs cross-linked by Ca^2+^. (**A**) Schematic outline of cross-linking alginate polymers by Ca^2+^ to obtain AH blocks. Light microscopy images of AH scaffolds visualized (**B**) parallel and (**C**) perpendicular to the aligned channels. (**D**–**E**) Cross-sectional view of the scaffold under the SEM. (**F**–**H**) SEM images of the channel walls. (**I**) Stress–strain curves of Ca^2+^ AHs tested parallel (P) or vertical (V) to the longitudinally aligned channels. Scale bar: (B–D and F) 100 μm; (E) 20 μm; (G) 50 μm; and (H) 1 μm.

AH scaffolds were fabricated by cross-linking alginate polymers with Ca^2+^ using as previously described [7]. Briefly, alginate sodium (Sigma) was completely dissolved in deionized water to obtain a concentration of 20 g/l alginate solution. After filtration (pore size, 0.2 mm; Millipore), 65 ml of the solution was transferred into a stainless-steel cylindrical mold (5 cm in diameter and6 cm height; LUYANG-Medical) and kept in a biosafety cabinet (Haier Biomedical) at room temperature (RT) for 6 h to remove air bubbles. Then, the mold was slightly tilted and rotated to attach the alginate solution to the wall and form a thin membrane on the surface. A sterile-filtered 1M CaCl_2_ (Sigma) solution was transferred into a humidifier (XunQiu Nano Mist) for atomization (1.25–1.45 ml/min) of the solution into ∼0.3-μm particles. The humidifier was placed 3 cm above the alginate solution spraying the alginate solution for 2 min until a visible gelated membrane occurred on the surface. Afterwards, 20 ml of CaCl_2_ solution was gently pipetted onto the alginate solution surface using a syringe over 3 min. The mold was covered with a lid and incubated in a biosafety cabinet for 36 h at RT. After removing the mold, the gelled AH was rinsed in deionized water four times (1.5 h each) to remove excessive electrolytes. Both ends of the AH with irregular capillaries were removed (Fig. 1A). The remaining middle portion of the hydrogel containing straight and parallel capillaries was stabilized through immersion in 0.1 M hexamethylene diisocyanate (Sigma) dissolved in dry acetone for 4 h on a shaker. Then, the hydrogel was washed with dry acetone for 10 min and placed on a filter paper to remove the remaining acetone. The stabilized AH was immersed in deionized water and heated to 75°C for 2 h until air bubbles no longer emerged from the hydrogel. The AH was rinsed in 0.1 M HCl (Sigma) eight times for 2 h to remove the cross-linking Ca^2+^ and stored in 75% ethanol. Separate batches of hydrogels were generated using ZnCl_2_ and CuCl_2_ instead of CaCl_2_.

### Evaluation of AHs mechanical properties

The AH stiffness was evaluated using an electromechanical universal testing machine (MTS Exceed E44, China) at RT. Hydrogels were cut into cubes of 10 mm × 10 mm × 10 mm and rinsed in Dulbecco’s phosphate-buffered saline (D’PBS) before testing. During compression, a speed of 0.1 mm/s was set to move for a total distance of 8 mm and data were recorded at a sampling rate of 50 Hz. Four samples of each AH were compressed, and results were averaged for evaluation. To obtain strain-stress curves, each hydrogel was tested in both orientations, parallel and vertical to the capillaries. To measure the elastic (Young’s) modulus, AH scaffolds (5 mm × 5 mm × 5 mm) were loaded on a mechanical testing device (Dynatek Dalta) and measured by pulling in tension parallel to the capillaries [26]. Five samples of each AH were analyzed.

### AH characterization

For additional structural analyses, hydrogels were cut into 0.5-mm-thick slices, rinsed in D’PBS for 2 h and placed on glass slides to obtain images at 100× magnification with a light microscope (Leica). Ten different images were taken for each hydrogel. Density, diameter and porosity of the capillaries were analyzed using ImageJ. The porosity was calculated as the percent area occupied by capillaries.

### Isolation of E14 rat spinal cord NSPCs

All animal experiments were performed following the guidelines approved by the Animal Ethical Committee of Huazhong University of Science and Technology. Female Sprague–Dawley (SD) rats (Vitalstar, Beijing) stably expressing green fluorescent protein (GFP) were used for NSPC isolation as previously described [27]. Embryos were removed from the uterus on embryonic day 14 (E14) after sacrificing. Embryonic spinal cords were dissected in D’PBS on ice and digested with0.25% trypsin (Gibco). A single-cell suspension was obtained post-filtration and cultured in neural basal medium containing a growth factor cocktail consisting of brain-derived neurotrophic factor (BDNF, 50 μg/ml, Abbkine), basic fibroblastic growth factor (10 μg/ml, Abbkine), vascular endothelial growth factor (10 μg/ml, Pepro Tech) and MDL28170 (50 μm, Apexbio), a cell-death inhibitor.

### *In vitro* culture and characterization NSPCs within AHs

Before NSPC seeding, AHs were cut into cubic scaffolds with a 2 mm × 2 mm × 3 mm dimension (length of capillaries: 2 mm) and washed three times in sterile D’PBS (2 h/wash). Scaffolds were placed in a dry 24-well culture dish with capillaries in a vertical orientation, and NSPC suspension (1 × 10^8^ cells/ml) was pipetted on the capillary openings. Cells were allowed to sink into the capillaries for 5 min to ensure that the entire length of the capillaries was filled with NSPCs before adding the neural basal medium with growth factor cocktails into the dish. AHs seeded with NSPCs were cultured in the medium for 1 day prior to transplantation.

For *in vitro* characterization, NSPCs and AHs cultured with NSPCs were fixed with 4% paraformaldehyde (PFA) for 20 min before rinsing with tris-buffered saline (TBS) at different time points. Samples were incubated with the following primary antibodies: rabbit anti-GFP (1:2000; ABclonal), mouse anti-Tuj-1 (1:2000; Santa Cruz), mouse anti-glial fibrillary acidic protein (GFAP, 1:1500; ABclonal), mouse anti-nestin (1:1000; Santa Cruz) and mouse anti-Olig2 (1:500; ABclonal). After washing with TBS, samples were incubated in secondary antibodies conjugated to Alexa Fluor 488 or 594. DAPI (0.25 μg/ml, Sigma) was used to stain cell nuclei.

### Characterization of AH and NSPC after seeding into AHs by scanning electron microscope

AHs with or without NSPCs were fixed with2% glutaraldehyde for 30 min and dehydrated by successive rinses in 30%, 50%, 70%, 90% and 100% alcohol (10 min each). After rinsing with tert-butanol for 5 min to remove alcohol, samples were soaked in *tert*-butanol and lyophilized in a freeze dryer (Boyikang) for 48 h, followed by coating with a 10-nm gold film. Microstructure images were taken using a Hitachi S-3000N scanning electron microscope (SEM) (Japan) with field emission set at 5 kV.

### Surgical procedures and corticospinal tract tracing

Adult wild-type SD rats weighing 140–160 g were used for *in vivo* examinations. Animals were randomly divided into three groups based on different grafts: AH loaded with NSPCs (AH-NSPC group, *n* = 37), AH without cells (AH group, *n* = 14) or NSPCs alone without alginate (NSPC-only group, *n* = 14). Rats in the AH-NSPC group were subdivided into five groups based on their survival time as indicated in Table 1. After intraperitoneal injection of chloral hydrate for anesthesia (250 mg/kg, Sinopharm), rats underwent a laminectomy at thoracic vertebra 8 (T8). The dura mater was longitudinally cut, and a 2-mm-long block of the spinal cord was completely removed through suction under a surgical microscope. Following hemostasis, the lesion was rinsed with 0.9% saline to remove debris and blood. For the AH-NSPC group, AHs cultured with NSPCs for 1 day were carefully implanted into the lesion cavities with capillary openings in close contact with both stumps of the spinal cord. Rats in the AH group were implanted with AH scaffolds without NSPCs after soaking in a culture medium for 1 day. For the NSPC-only group, cells were collected from the dish 1 day after culture and resuspended in a neural basal medium with growth factor cocktail at a concentration of 25 000 cells/μl. A total of 5 μl of cell suspension were slowly injected into the lesion cavities. The total number of grafted NSPCs was approximately the same number of cells as in AHs. Following grafting, the dural openings were tightly covered with an artificial dura mater (Integra) to prevent leakage of grafted cells. The overlying muscles and skin were sutured [7]. Animal care was performed as previously described [13], and bladders were manually emptied twice daily up to 2 weeks until spontaneous micturition recovered.

**Table 1.** Experimental groups

Animal survival	Grafts	# of animals			Total animals
		Histological analysis	Electrophysiological study	BBB scoring	
0 weeks^a^	AHs-NSPCs	5		5	5
2 weeks	AHs-NSPCs	6		6	6
4 weeks	AHs-NSPCs	6		6	6
6 weeks	AHs-NSPCs	6		6	6
8 weeks	AHs-NSPCs	7	7	7	14
8 weeks	Ahs	7	7	7	14
8 weeks	NSPCs	7	7	7	14

a Animals were perfused 1 day postoperatively to evaluate total cell loading in the hydrogels.

Two weeks before sacrifice, a separate rat cohort (*n* = 7) was randomly selected for corticospinal tract (CST) tracing [7]. Rats were fixed in a stereotaxic apparatus following anesthesia and injected with 10% biotinylated dextran amine (BDA; MW 10 kDa; Invitrogen) at the following coordinates from Bregma: anteroposterior (AP) +0.5, mediolateral (ML) ±2.0; AP ±1.0, ML ±1.0; AP −1.5, ML ±2.0; and AP −1.0, ML ±3. Approximately 1 μl of BDA was injected at each site at a depth of 1.2 mm. To exclude a possible influence of tracing surgeries on motor performance, these animals did not undergo behavioral testing during the 2 weeks following BDA injections.

### Electrophysiological monitoring and behavioral assessment

Electrophysiological conductivity was recorded using a previously reported method [7] with minor modification. Seven rats were randomly chosen from each group for terminal electrophysiological examinations on the day of perfusion. Following anesthesia, spinal cord segments for electrode placement were exposed. Stimulation electrodes for delivering a square-wave pulse (400 Hz, 0.8 mA) were inserted into the cord at the T3 level while recording electrodes placed at the T10 level. Each rat received >20 consecutive stimulations with an interval of 2 s and a pulse duration of 200 μs. All electrodes were connected to a 32 XLTEK MEP system (Natus) for stimulation and recording. The maximal response of all stimulations and its relative latency for each rat were calculated for further analysis.

For behavioral tests, the 21-point Basso, Beattie, Bresnahan (BBB) locomotor rating scale [28] was used to assess the locomotor function of the hindlimbs by two observers blinded to group identity. Rats were placed in an open enclosure (150 cm diameter), observed for 3–5 min and assessed 1 day pre-operatively as the baseline and then once a week thereafter post-operatively. The scores of both hindlimbs were averaged [7].

### Tissue processing and immunohistochemistry

Rats were deeply anesthetized and transcardially perfused with ice-cold phosphate-buffered saline followed by ice-cold 4% PFA in 0.1 M phosphate buffer (PB). The spinal cords were dissected, postfixed in 4% PFA for 1 h and then transferred into 30% sucrose in 0.1 M PB for cryoprotection. For histological analysis, a 2.5-cm-long spinal cord segment centered on the lesion/graft site was cut into 25-μm horizontal sections on a cryostat. Sections were consecutively collected and directly mounted in 14 sequential series on glass slides.

For immunohistochemical staining, the sections were rinsed with TBS and then blocked with 5% horse serum/0.25% Triton X-100 in TBS (TBST), followed by incubation with primary antibodies diluted in 1% donkey serum in TBST overnight. After rinsing with TBS, sections were incubated with species-specific secondary antibodies coupled to Alexa Fluor-488, -594 or -Cy5, and DAPI to label nuclei. Sections were rinsed in TBS and coverslipped with Fluoromount G (Southern Biotech) after dehydration. The following antibodies were used: rabbit anti-GFP (1:1000; ABclonal), mouse anti-Tuj-1 (1:1000; Santa Cruz), mouse anti-glial fibrillary acidic protein (GFAP, 1:1000; ABclonal), mouse anti-NeuN (1:1000; Santa Cruz), mouse anti-nestin (1:800; Santa Cruz), mouse anti-Olig2 (1:500; ABclonal), rabbit anti-synaptophysin (Syn, 1:500; Dako) and streptavidin-Alexa 594 for BDA-traced axons (1:700; Jackson Immuno Research). Images were taken using a MicroPublisher 6 (Canada) system connected to an Olympus BX51 microscope.

### Quantification of grafted cells within the lesion and their extending neurites in the host cord

To quantify the grafted NSPCs within the hydrogel or lesion, 1 out of 14 serial sections was double-labeled with GFP and differentiation markers including GFAP, Tuj-1 and Olig2. Cells positive for both markers were counted, and the number was multiplied by 14 to obtain the total number of cells in the AH graft.

To quantify axonal profiles extending from the grafts, imaginary lines in the rostral and caudal parenchyma were set at specific distances to the lesion border marked by GFAP labeling. GFP (+) neurites crossing these lines were quantified in 1 of 14 serial sections.

### Quantification of host regrowing axonal profiles within the AHs

Host axonal profiles within AH channels were quantified as previously described [7, 13]. Briefly, imaginary lines perpendicular to the channels were set between the rostral edges of the AHs under the microscope at 20× magnification. Tuj-1 (+) but GFP (−) axons in all channels crossing each line were identified as regrowing host axons in 2 out of 14 section series. The following equation was used to calculate the number of axons per mm^2^ AH grafts:

$$N=\frac{1\;000\;000\times \sum \mathrm{axons}}{\mathrm{section}\;\mathrm{thickness}(\mu m)\times \sum \mathrm{width}\;\mathrm{of}\;\mathrm{the}\;\mathrm{hydrogel}\;\mathrm{at}\;a\;\mathrm{specific}\;\mathrm{distance}(\mu m)}.$$

All quantifications were performed by an observer blinded to group identity.

### Statistical analysis

All data are expressed as mean ± stand error of the mean and analyzed using GraphPad Prism 5.01 software. Between-group comparisons were performed using unpaired Student’s *t*-test or non-parametric analysis for non-normally distributed data. One-way analysis of variance (ANOVA) with Tukey’s *post hoc* tests or two-way ANOVA with Bonferroni *post hoc* tests were used for multiple comparisons. A *P*-value of <0.05 was considered statistically significant.

## Results

### Characterization of AHs cross-linked by Ca^2+^

The diameter of anisotropic capillaries within the hydrogel was 178.9 ± 8.8 μm (Fig. 1B and C), which was larger than hydrogels cross-linked by Zn^2+^ (105.8 ± 15.7 μm) and Cu^2+^ (55.2 ± 5.3 μm) (supplementary Fig. S1A) fabricated using the same method but different electrolytes. However, the capillary density of Ca^2+^ alginate scaffolds was 31 ± 2 channels/mm^2^, which was lower than that of Zn^2+^ and Cu^2+^ alginate scaffolds (supplementary Fig. S1B). The channel porosity of Ca AH (68.4 ± 6.5%) was slightly higher than hydrogels cross-linked by Zn^2+^ (66.2 ± 9.0%) and Cu^2+^ (62.5 ± 5.3%) (supplementary Fig. S1C). Ultrastructural SEM imaging further demonstrated evenly distributed linear channels in the hydrogels. The inner wall of the capillaries was not completely smooth but contained a coarse surface with randomly distributed protrusions (Fig. 1D–H). The curves of stress-strain tested in different directions are shown in Fig. 1I. While compressing the hydrogels in a direction parallel or vertical to the capillaries, the stress exerted on Ca^2+^ AHs was lower compared to Zn^2+^ and Cu^2+^ AHs (supplementary Fig. S1D). The elastic modulus was 226.0 ± 11.3 kPa, which is insignificantly higher than that of Zn^2+^ AHs (215.4 ± 11.0 kPa) but lower than Cu^2+^ AHs (235.0 ± 10.3 kPa) (supplementary Fig. S1E).

### Characteristics of spinal cord NSPCs in AHs after an *in vitro* culture

**Figure 2.**
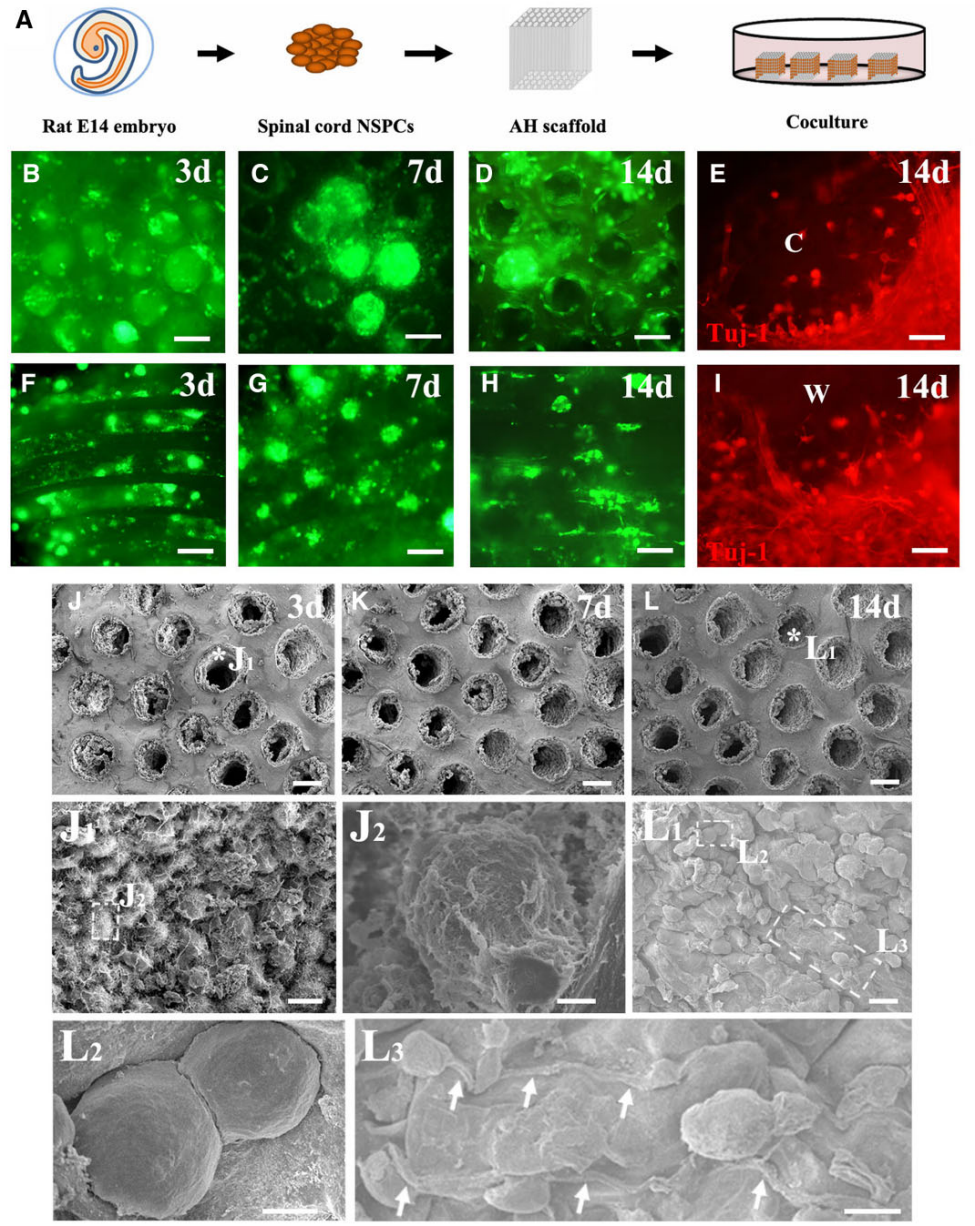
Characteristics of NSPCs within AH scaffolds cultured *in vitro*. (**A**) Schematic illustration of NSPC isolation from E14 rat spinal cord and seeding into AH scaffolds. (**B–D**) GFP-positive NSPCs within the AH scaffolds cultured for 3, 7 and 14 days visualized from the channel openings. (**E**) Cross view of NSPCs cultured in the channel for 14 days immunolabeled with Tuj-1 (red; c: channel lumen). (**F–H**) NSPCs within the AH scaffolds cultured for 3–14 days visualized parallel to the channel direction. (**I**) Longitudinal view of NSPCs cultured in the channel for 14 days immunolabeled for Tuj-1 (red; w: channel wall). (**J–L**) View of AH channels filled with NSPCs after culture for 3, 7 and 14 days *in vitro* under the SEM. (**J_1_**) Higher magnification ultrastructural SEM image of the point labeled with a star in (J). (**J_2_**) Higher magnification of the boxed area in (J_1_). (**L_1_**) Higher magnification of the area labeled with a star in (L). Boxed areas are magnified as (**L_2_**) and (**L_3_**). NSPCs extend long processes (arrows) along the channel wall. Scale bar: (B–D and F–H) 100 μm; (E and I) 20 μm; (J–L) 100 μm; (J_1_ and L_1_) 20 μm; (J_2_ and L_2_) 4 μm; and (L_3_) 10 μm.

Three days after AH seeding (Fig. 2A), NSPCs proliferated and formed spheres (Fig. 2B and F) similar to those cultured in dishes with the same medium without AHs (supplementary Fig. S2A). Most NSPC spheres were located in the middle of the channels with a thin layer of cells attaching to the wall (Fig. 2B–D and F–H). These findings were also clearly observed when using SEM to examine the NSPC ultrastructure in AHs (Fig. 2J, J_1_ and J_2_). NSPCs were round even 7 days after seeding in the channels (Fig. 2C, G and K). Only few NSPC spheres were seen after a cultivation for 2 weeks in the scaffold (Fig. 2D and H). Round NSPCs became flat and adhered to the wall (Fig. 2L1 and L_2_) whereas and some irregularly shaped cells extended single or multiple processes (Fig. 2L3). These processes were predominantly Tuj-1 (+) (Fig. 2E and I), indicating that NSPCs differentiated into neurons within the channels. Only few cells were labeled with GFAP (data not shown).

**Figure 3.**
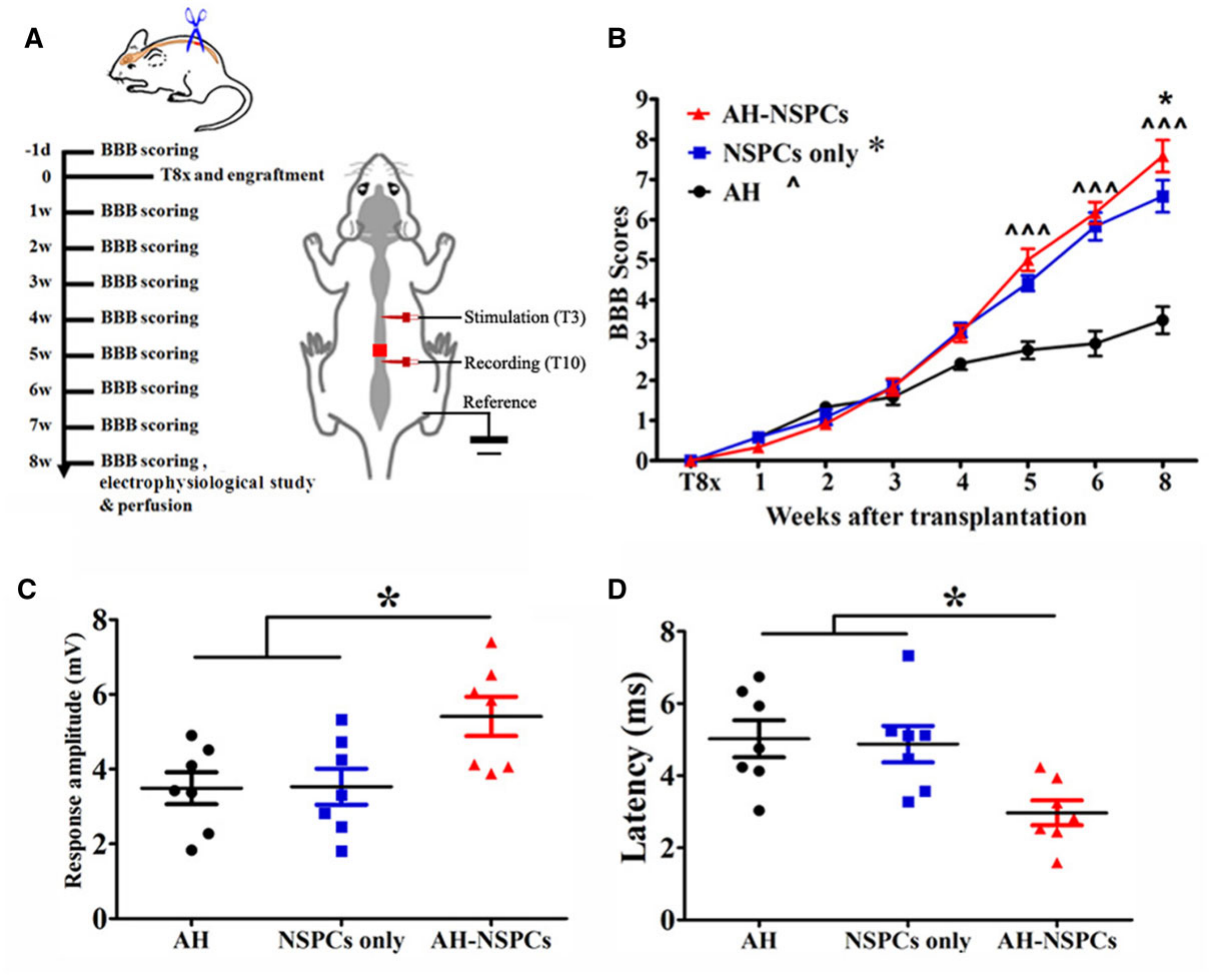
Locomotor recovery and electrophysiological restoration after AH scaffold and NSPC transplantation. (**A**) Timeline of the experiment and schematic illustration of electrophysiological assessment. (**B**) BBB open-field locomotor scores of each group (*P <* *0.05, AH-NSPC *vs* NSPC group; ^^^*P *<* *0.001, AH-NSPC and NSPC groups *vs* AH group; two-way ANOVA followed by Bonferroni *post hoc* test). Animals grafted with AH-NSPCs showed a (**C**) higher response amplitude and (**D**) shorter latency caudal to the lesion after stimulation rostral to the lesion site (*P <* *0.05; one-way ANOVA followed by Tukey’s *post hoc* analysis).

### NSPC-seeded AHs for locomotor and electrophysiological recovery

After the T8 transection injury, all subjects showed complete paralysis of their hindlimbs (BBB scores = 0). BBB scores were not significantly different between animals receiving different grafts during the initial 4 weeks post-injury. However, starting at 5 weeks post-injury and transplantation, BBB scores of subjects receiving either NSPCs or AH combined with NSPCs were significantly higher than scores of animals transplanted with AHs without cells. Eight weeks post-injury, animals grafted with AHs and NSPCs showed even higher BBB scores than the NSPC cohort (Fig. 3A and B).

Electrophysiological monitoring indicated that animals in the AH-NSPC group had greater response amplitudes (5.4 ± 0.5 mV, *P* = 0.016) than those in both AH (3.5 ± 0.4 mV) and NSPC-only (3.5 ± 0.5 mV) groups (Fig. 3C). The response latency in the AH-NSPC group was 3.0 ± 0.3 ms, which was shorter than that detected in AH (5.0 ± 0.5 ms) and NSPC-only (4.9 ± 0.5 ms) groups (*P *=* *0.010; Fig. 3D). These results further support a partial functional restoration of the spinal cord by AH and NSPC co-transplantation.

### NSPC-seeded AHs for histological restoration of the injured spinal cord

**Figure 4.**
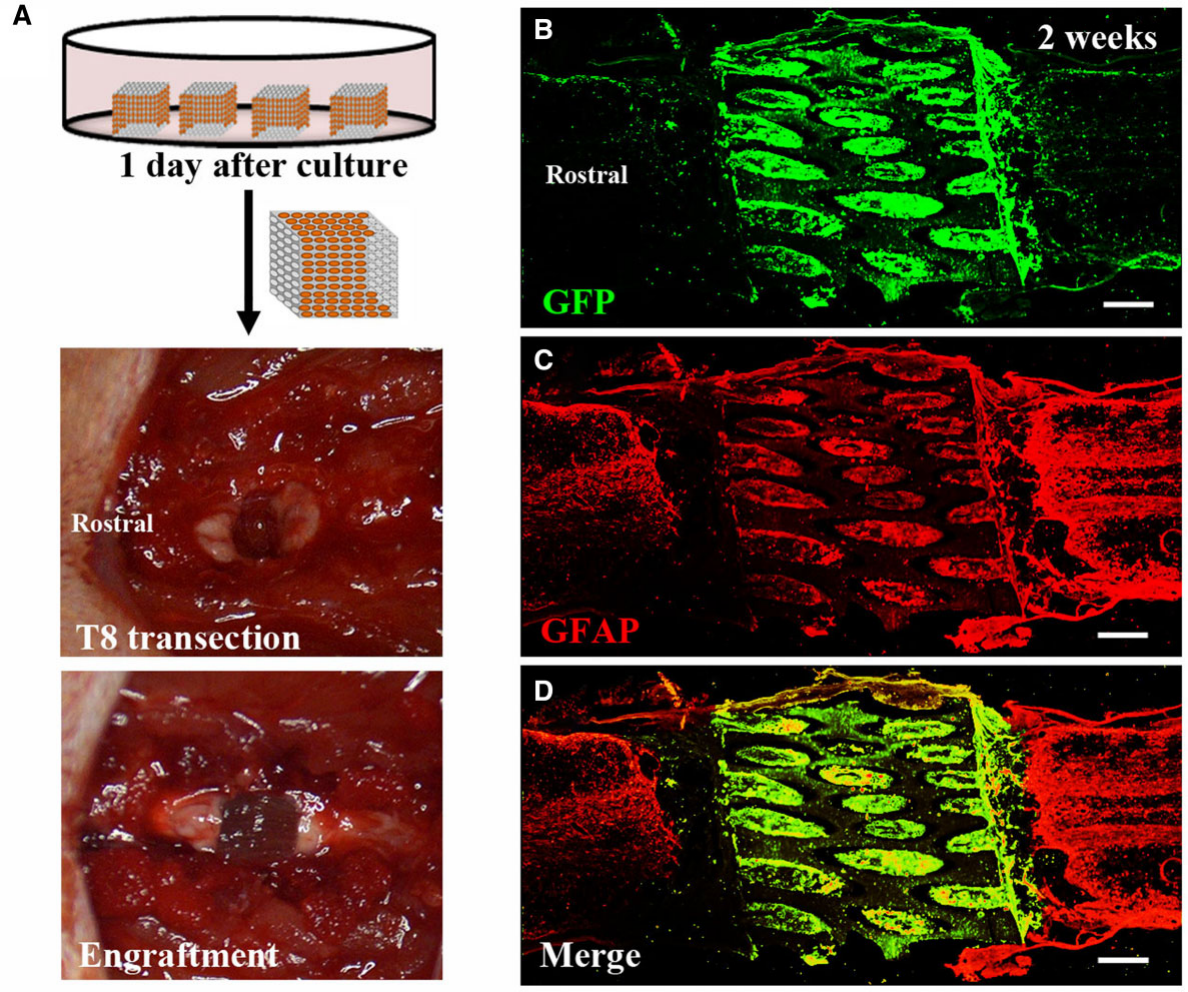
Transplantation and integration of AH scaffolds combined with NSPCs in the injured spinal cord. (**A**) Surgical procedure for implantation of NSPC-loaded AHs into the spinal cord lesion. (**B**–**D**) Horizontal sections of the lesion site 2 weeks after engraftment shows alginate scaffold filled with GFP-labeled NSPCs (green) connecting both stumps of the host spinal cord identified by GFAP labeling (red). Scale bar: (B–D) 500 μm.

Immunohistochemical labeling indicated that NSPC-seeded AHs completely filled the lesion and restored the spinal cord continuity. A mild hypercellularity of astrocytes labeled with GFAP was observed in both stumps of the host tissue (Fig. 4). Cavitations were scarce between the graft and host spinal cord. In contrast, in subjects grafted with NSPCs only, cavitations were found in the lesions (3/7 animals), and atrophy of both stumps was commonly observed (4/7 animals) (supplementary Fig. S3A and B).

### Fate of NSPCs within AHs at post-transplantation in SCI

**Figure 5.**
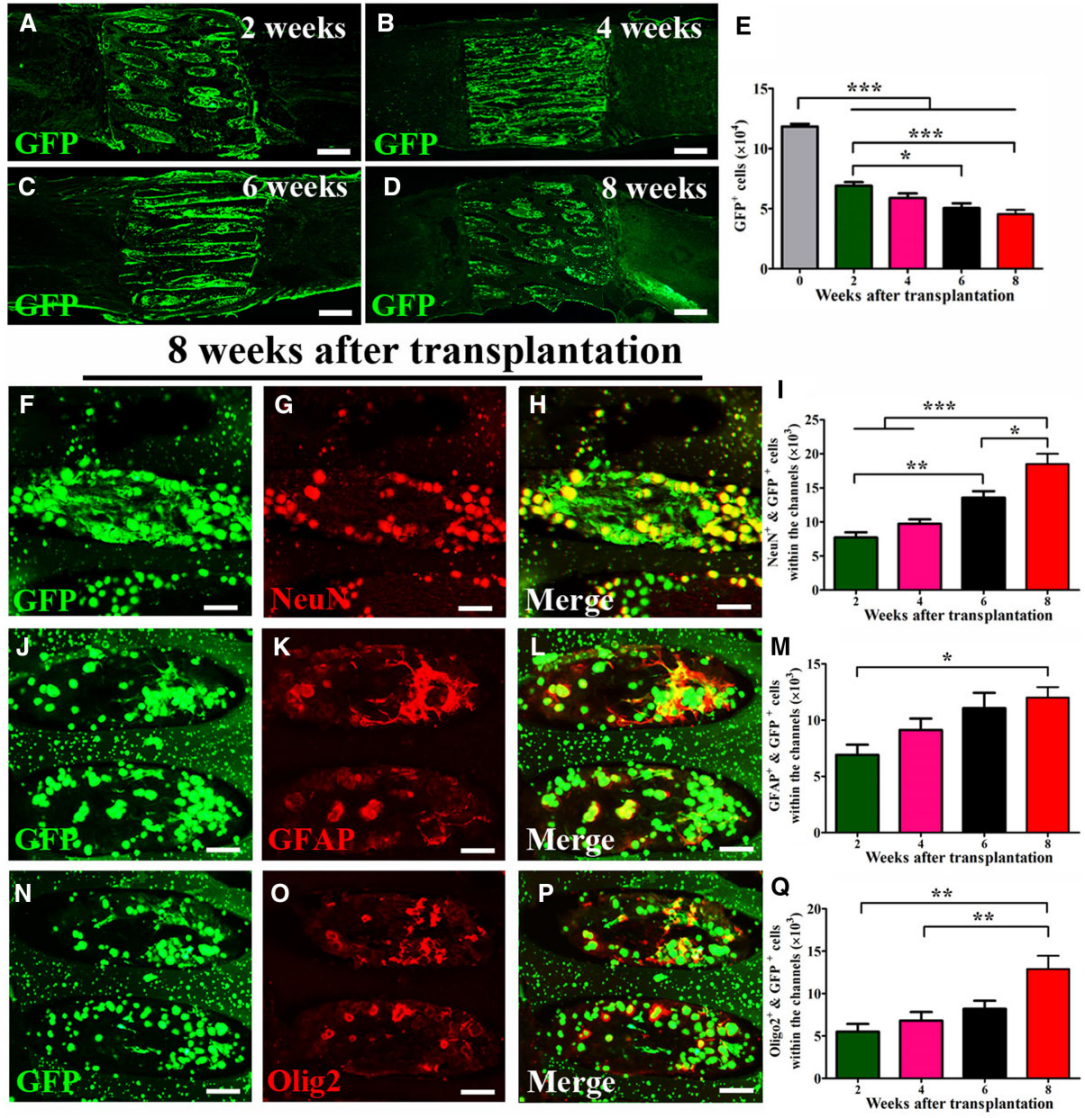
Fate of NSPCs within AH channels post-transplantation. (**A–D**) GFP-labeled NSPCs (green) survived within and around the AH scaffold post-implantation. (**E**) Quantification of GFP (+) cells within the channels at different time points. (**F–H**) NSPCs within the channels differentiated into mature neurons labeled for NeuN (red). (**I**) Quantification of differentiated neurons (GFP/NeuN double-labeled) at different time points. (**J**–**L**) NSPCs (green) in the channels differentiated into astrocytes were identified by GFAP labeling (red) at 8 weeks post-transplantation. (**M**) Quantification of differentiated astrocytes (GFP/GFAP double-labeled) at different time points. (**N–P**) Grafted NSPCs also differentiated into oligodendrocytes labeled for Olig2 (red) at 8 weeks post-transplantation. (**N**) Quantification of differentiated oligodendrocytes (GFP/Olig2 double-labeled) at different time points (*P <* *0.05; **P <* *0.01; ***P <* *0.001; one-way ANOVA followed by Tukey’s *post hoc* analysis). Scale bar: (A–D) 500 μm and (F–H, J–L and N–P) 50 μm.

GFP expressing NSPCs survived in the scaffolds and some cells migrated into the graft/host interface. A few GFP (+) cells could be observed in the host parenchyma (Fig. 5A–D). The number of grafted cells within channels decreased over time, and more than one-third (38.3 ± 3.0%) of GFP (+) cells were detected 8 weeks post-transplantation (Fig. 5E). A sharp decrease in cell number occurred in the first 2 weeks post-engraftment with further declines at 6 and 8 weeks. Over time, differentiation and maturation of NSPCs was observed in the channels (Fig. 5I, M and Q). NSPCs predominantly differentiated into neurons (immunolabeled for NeuN, Fig. 5F–I), astrocytes (immunolabeled for GFAP in Fig. 5J–M) and oligodendrocytes/oligodendrocyte precursors (immunolabeled for Olig2 in Fig. 5N–Q). The number of differentiated NSPC-derived cells within the scaffolds continuously increased, and the number of all three types of differentiated cells was significantly higher at 8 weeks compared to the 2 week time point. At 8 weeks post-implantation, differentiation ratios of neurons, astrocytes and oligodendrocytes relative to the total number of surviving grafted cells within the scaffolds were 40.7 ± 3.9%, 26.6 ± 1.8% and 28.4 ± 1.7%. Nestin (+) cells were rarely detected, neither in the scaffold nor the host/graft interface (data not shown).

**Figure 6.**
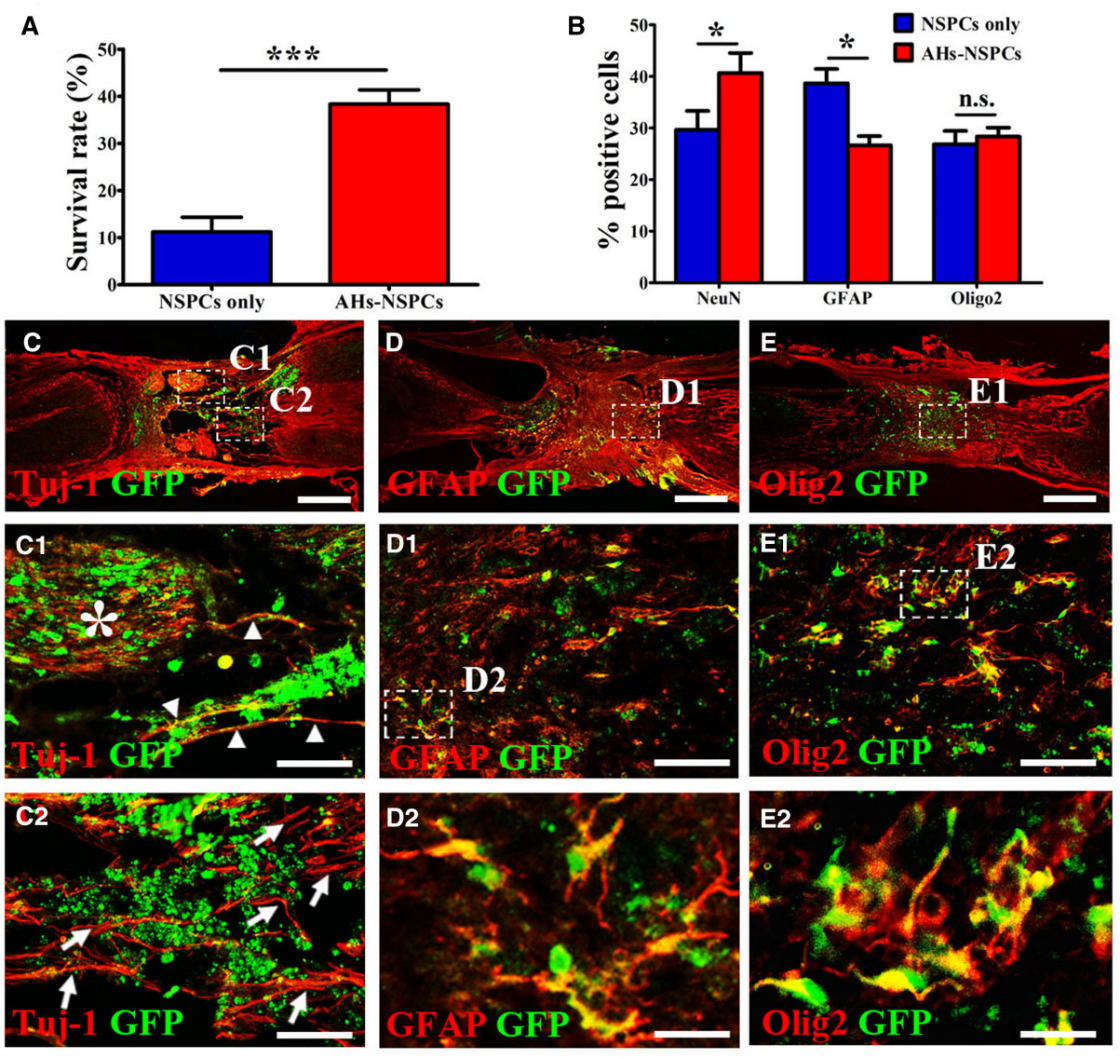
Fate of NSPCs in the lesion and effects of NSPCs on axonal regrowth in NSPC-grafted animals without AHs. (**A**) Comparison of NSPC survival in hydrogels/lesion site between the NSPC-only and AHs-NSPC groups at 8 weeks post-engraftment (***P <* *0.001, unpaired Student’s *t*-test). The percentage of surviving cells in the NSPC group was calculated as the proportion of the total number of grafted cells (1.25 × 10^5^ cells). (**B**) Comparison of cell fates between the NSPC-only and AHs-NSPC groups for neurons, astrocytes and oligodendrocytes (n.s., *P* > 0.05, *P <* *0.05, unpaired Student’s *t*-test). (**C**) Grafted NSPCs (green) differentiated into neurons (co-labeled forTuj-1) and supported host axonal regeneration (red only) in the lesion. (**C_1_** and **C_2_**) Higher magnifications of the boxed area in (**C**) shows axons sprouting from grafted NSPCs (arrowheads) and host neurons (arrows and area marked with a star) arranged in a randomized pattern. (**D**) NSPCs (green) partly filled the lesion and differentiated into astrocytes (co-labeled for GFAP). Cavitations associated with atrophic stumps were observed around the lesion. (**D_1_**) is a higher magnification of the boxed area in (**D**), and (**D_2_**) is a higher magnification of the boxed area in (**D_1_**) to better visualize the differentiated astrocytes. NSPCs (green) differentiated into oligodendrocytes (co-labeled for Olig2). (**E_1_**) is a higher magnification of the boxed area in (**E**), and (**E_2_**) is a higher magnification of the boxed area in (E_1_) to show differentiated oligodendrocytes. Scale bar: (C–E) 500 μm; (C_1_, C_2_, D_1_ and E_1_) 100 μm; and (D_2_ and E_2_) 20 μm.

An even more profound decline in the overall NSPC survival rate (11.2 ± 3.1%) at 8 weeks post-transplantation was evident in animals grafted with NSPCs without scaffolds. This was significantly lower compared to NSPC survival in animals with NSPCs seeded into AHs (Fig. 6A). Even though the differentiation ratio of oligodendrocytes in the NSPC-only cohort was comparable to that of the AH-NSPC group, NSPCs without AHs co-transplantation had a lower proportion of neurons (29.7 ± 3.7%) but a higher proportion of astrocytes (38.7 ± 2.8%) compared to cell differentiation in the AH-NSPC group (Fig. 6B–E and C_1_–E_2_).

### Seeding of NSPCs increases axonal regeneration into the alginate scaffolds

**Figure 7.**
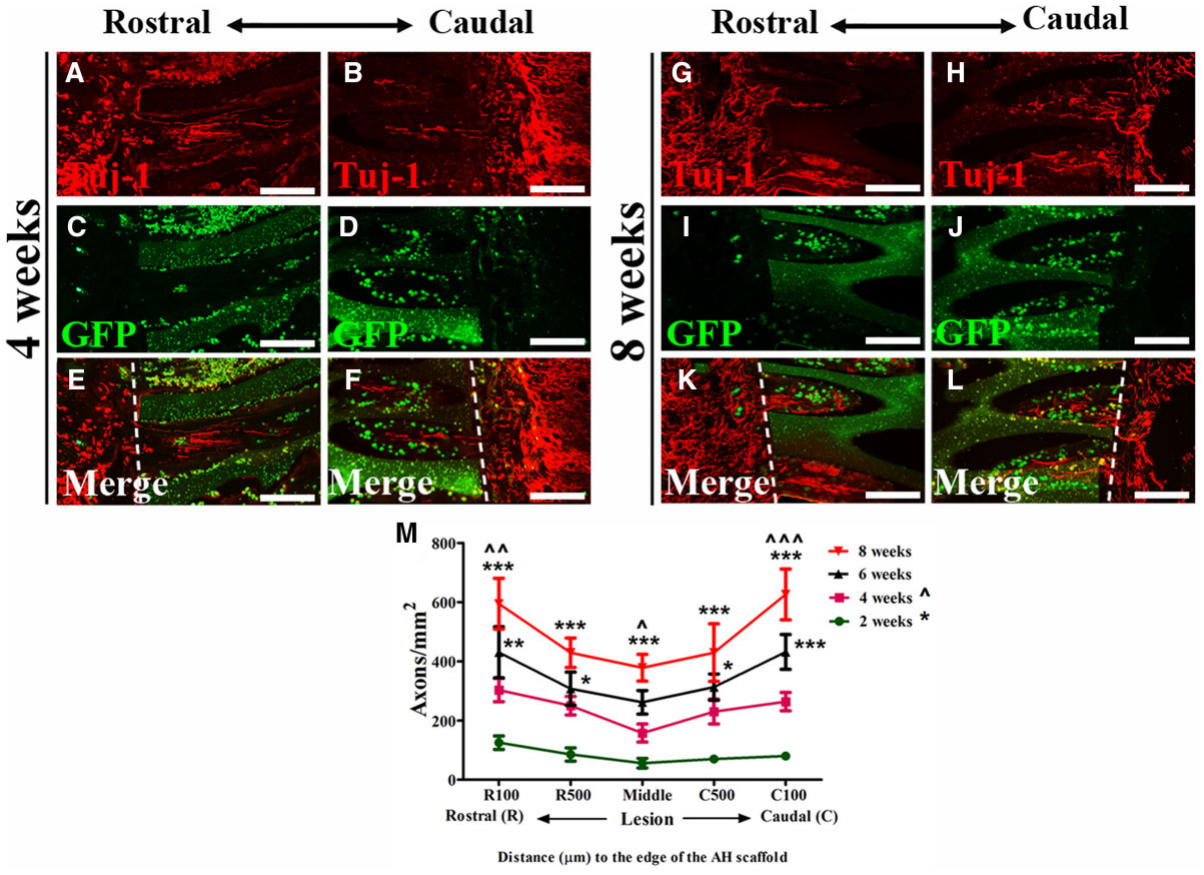
Regeneration of host axons after combined NSPC and AH scaffold transplantation. (**A**–**F**) Tuj-1 (red)-labeled axons at the rostral and caudal areas of the hydrogel elongate along the NSPC-filled (green) channels at 4 weeks post-engraftment. (**G–L**) An increased number of Tuj-1-labeled (red) axons are found within the channels at the rostral and caudal areas of the hydrogel at 8 weeks post-engraftment. Dashed lines in (**E**–**F**) and (**K**–**L**) indicate hydrogel edges. (**M**) Quantification of Tuj-1 (+) but GFP (−) axons within AH capillaries at specific distances from the hydrogel/host interface in animals perfused at different time points (*P <* *0.05, **P <* *0.01, ***P <* *0.001 *vs* animals perfused at 2 weeks post-transplantation; ^*P *<* *0.05, ^^*P *<* *0.01, ^^^*P *<* *0.001 *vs* animals perfused at 4 weeks post-transplantation; two-way ANOVA followed by Bonferroni *post hoc* test). Scale bar: (A–L) 100 μm.

To explore the effects of NSPC-filled AHs on host axonal regeneration, sections were immunolabeled with Tuj-1 and GFP. A large amount of Tuj-1 (+)/GFP (−) regrowing axons approached the graft/host interface and entered the channels in a linear pattern (Fig. 7A–L). This growth could be observed as early as 2 weeks post-transplantation. Axons extended across the full length of NSPC-filled channels with a slight decrease in the number of neurites toward the center of the scaffolds. The number of axons significantly increased at all distances from the host/scaffold interface over time with a more than 3-fold increase at 8 weeks compared to 2 weeks post-grafting (Fig. 7M). In subjects transplanted with AHs without cells, only a few axons could be observed near the interface, and only a sparse number of axons could be detected in the channels even 8 weeks post-transplantation. Host axonal regeneration could also be found in NSPC grafts without AHs but axons were oriented in a more randomized pattern (Fig. 6C, C_1_ and C_2_).

### Axonal extension of grafted NSPCs and reciprocal synapse formation with host neurons

**Figure 8.**
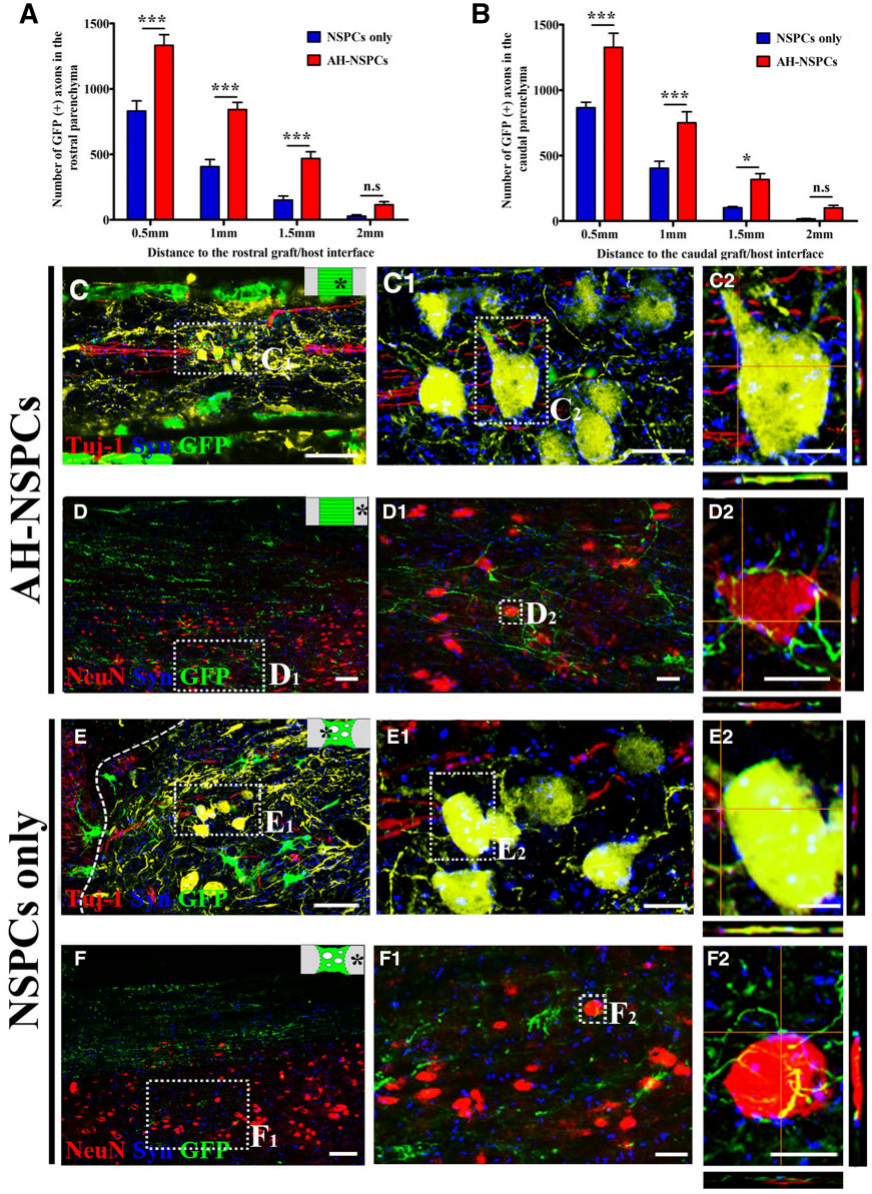
Axonal extension from NSPC grafts and reciprocal synaptic connectivity with host neurons at 8 weeks post-transplantation. (**A** and **B**) Quantification of axons from NSPCs in the rostral and caudal host spinal cord at different distances from the lesion border (*P <* *0.05, ***P <* *0.001; two-way ANOVA followed by Bonferroni *post hoc* test). (**C**) Host axons (red) extend within the AH channel filled with NSPCs (green). (**D**) Numerous NSPC-derived axons (green) grow out of the AHs and extend in the host cord. (**E**) Host axonal regeneration (red) in the lesion grafted with NSPCs (green). the dashed line indicates the graft/host interface. (**F**) Outgrowth of axons (green) from the NSPC graft into the host parenchyma. (**C_1_**–**F_1_**) Higher magnification of the boxed areas in (C)–(F). (**C_2_** and **E_2_**) Higher magnification of the boxed area in (C_1_) and (E_2_) shows host axonal terminals colocalized with the presynaptic marker syn in close contact with neurons differentiated from grafted NSPCs (green). (**D_2_** and **F_2_**) Higher magnification of the boxed area in (D_1_) and (F_1_). NSPC-derived axonal terminals co-labeled for Syn were found in close apposition to host neurons (red). Graphic insets in the upper right corner of (C)–(F) indicate the imaged area. Scale bar: (C–F) 40 μm; (C_1–_F_1_) 10 μm; and (C_2_–F_2_) 5 μm.

Axons derived from grafted NSPCs identified by Tuj-1/GFP co-localization also grew into the host spinal cord at 8 weeks post-transplantation. Quantification of GFP (+) axons indicated that significantly more graft-derived axons extended into the host spinal cord of AH-NSPC-grafted animals compared to those animals that received only NSPCs. Axon growth was similar in rostral and caudal direction and significantly higher for a distance up to 1.5 mm. Rare axons were found in the host parenchyma at 2.5 mm beyond the lesion border marked by GFAP labeling (Fig. 8A and B).

For both AH-NSPC and NSPC groups, GFP-labeled axons mostly extended in the host white matter in organized and linear patterns. Some GFP (+) projections sprouted lateral branches into the gray matter and formed punctuate contacts with host neurons immunolabeled by NeuN (Fig. 8D, D_1_, D_2_, F, F_1_ and F_2_). These contacts were also colocalized with the presynaptic marker synaptophysin (Syn), suggesting possible synaptic connections. In grafts with NSPCs, bouton-like terminals were also observed on host axons colocalized with Syn and were closely associated with neurons differentiated from grafted NSPCs (Fig. 8C, C_1_, C_2_, E, E^1^ and E_2_). These findings support the presence of reciprocal graft–host synaptic connections.

## Discussion

This study developed AH scaffolds cross-linked by Ca^2+^ and consisting of parallel channels with an average size of 178.9 ± 8.8 μm for transplantation in the completely transected rat spinal cord. Our data indicate that Ca scaffolds have larger capillary diameters than those cross-linked by Zn^2+^ and Cu^2+^ consistent with previous studies [6–8, 13, 29]. In vitro cultivation of NSPC in Ca^2+^ scaffolds show NSPC survival and differentiation into neurons. Upon grafting NSPC-loaded AH scaffolds into a T8 spinal cord transection, NSPCs within AHs differentiated into neurons and glia, supported robust host axonal regeneration into the channels and extension of axons from graft-derived neurons into the host spinal cord. Importantly, graft-derived axons formed putative synaptic contacts with host neurons. Reciprocally, host axon terminals labeled for synaptic proteins were juxtaposed to graft-derived neurons. These findings support the formation of neuronal relays across the lesion to promote partial electrophysiological and functional recovery.

Cross-linking AHs by different divalent cations has been widely studied in tissue engineering with Ca^2+^ being the most used ion [30–35]. For myocardial infarction and heart failure repairs, alginate subunits can be spontaneously chelated by Ca^2+^available in the surrounding tissues [36]. In the spinal cord with more limited amounts of Ca^2+^ in the extracellular matrix, AHs can be formed by co-injecting alginate and Ca^2+^ solution [11]. *In situ* gelling processes result in isotropic pore structures within hydrogels [37]. AHs were therefore generated prior to implantation and excess Ca^2+^, which may result in synaptic dysfunction, impaired plasticity and neuronal cell death [38] was removed after cross-linking to prevent possible adverse effects. AHs generated with Ca^2+^ were stable *in vitro* and *in vivo* for at least 8 weeks consistent with previous studies using other divalent cations [6, 7, 13, 14].

AH scaffolds with anisotropic capillaries have been investigated in several studies as a potential biomaterial for SCI repair [6, 7, 13, 14, 29]. In the current and our previous studies, AHs showed excellent biocompatibility and AH transplantation alone can provide continuity of the transected spinal stumps, thereby supporting the regeneration of axons [7]. The number of regenerating axons can be remarkably enhanced by filling channels with BMSCs [6], SCs [13] or neonatal astrocytes [14]. Increased BDNF levels overexpressed by seeded BMSCs [6] or virus injected caudal to the lesion [13] further promote host axonal regeneration in AHs. In the present study, E14 NSPCs survived within the channels for at least 8 weeks and regeneration of a large number of host axons extending in a linear pattern similar to the white matter of the spinal cord was promoted by NSPCs. Collectively, AHs cross-linked by different divalent cations can serve as effective carriers for different cells and physical guidance for axonal regeneration.

Transplantation of NSPCs has several advantages for SCI repair compared to grafts of other cells due to the multipotency of NSPCs. Mechanisms underlying functional restoration after NSPC transplantation to the injured spinal cord include neuroprotection, immunoregulation, remyelination and formation of neuronal relays for reconnection of the disrupted neural circuitry [25]. In this study, we also observed that graft-derived axons extensively reinnervated the host spinal parenchyma extending for up to 2 mm similar to previous reports [39]. NSPC-derived neurons formed reciprocal synaptic connections with host neurons providing the basis for neuronal relays across the lesion site. Such new relay circuits might be an important factor contributing to the observed electrophysiological restoration and functional improvement [39].

In addition to neuronal differentiation and axonal outgrowth by NSPC-derived neurons, numerous host axons extended into the NSPC-filled microchannels. The number of regrowing host axons increased over time indicating that NSPCs continuously facilitate host axonal growth. Despite robust host axonal growth into AHs filled with NSPCs (Fig. 7), CST axons did not penetrate channels with grafted cells (supplementary Fig. 3). Regeneration of CST axons into NSPCs is dependent on injury, direct contact between NSPCs and CST axons, the presence of neurons in grafted cells, and is further enhanced by a homotypic spinal origin of cells [24]. In the current study, homotypic grafts were used and CST axons were completely transected. However, placement of NSPC-filled AHs into the lesion site might not be sufficient to bring severed CST axons in direct contact with grafted cells, thereby limiting CST responses. Additional injections of a small number of NSPCs at the rostral host/graft interface might be one means to promote CST regeneration. In addition to neuronal differentiation, glial differentiation of NSPC-derived astroglia might play an equally important role in enhancing overall host axonal growth as previously shown for AHs filled with neonatal astrocytes [14]. Finally, myelination by differentiating oligodendrocytes might contribute to the stabilization of host and graft-derived axons [5].

Direct injection of cells in the lesion site is one of the most common approaches for cellular grafting after SCI [4]. However, cell suspensions can be easily flushed out by the cerebrospinal fluid failing to completely bridge the lesion [40]. The use of three-dimensional scaffolds can effectively confine cells in the lesion cavity [4]. In this study, surviving cells were primarily located within the channels. NSPCs were rarely observed on the cord surface distal to the lesion and only a small proportion of NSPCs was found around the AH scaffold. These cells might have been “squeezed out” by handling AHs during implantation or alternatively, NSPC might have migrated into the surrounding host tissue. NSPCs have been found to migrate out of the graft/lesion site along white matter tracts of the host parenchyma both rostrally and caudally for up to 15 mm beyond the lesion site [4]. However, in other studies using rat [39] or human [23] NSPCs, grafted cells were not observed in the host cord beyond the immediate region of the graft/lesion site. NSPCs located in close proximity to the scaffold could facilitate integration of AH with the host spinal cord making the graft/host interface more permissive for axonal penetration [13].

The microenvironment within a scaffold might affect NSPC differentiation. In a study using 3D-printed polyethylene glycol–gelatin methacrylate scaffolds loaded with freshly dissociated E14 spinal cord NSPCs, ∼% of the grafted cells expressed the neuronal marker NeuN, 21% expressed the astrocyte marker GFAP and 11% expressed the oligodendrocyte marker Olig2 at 1 month post-engraftment [5]. Grafting cells with a 2-week delay post-injury in a complete T3 injury, ∼28% of grafted NSPCs differentiated into NeuN-labeled neurons, 16% into astrocytes and 27% into oligodendrocytes 6 weeks post-transplantation [39]. In this study, differentiation rates were compared between the NSPC-only and AHs-NSPC groups at 8 weeks post-implantation, indicating that the AHs-NSPC group had a higher neuronal differentiation rate (40.7%) than the NSPC-only group (29.7%). Higher neuronal differentiation was accompanied by reduced astrocytes differentiation (26.6% *vs* 38.7% in the NSPC-only group) suggesting that AHs facilitates differentiation of NSPCs into neurons.

Scaffolds have been reported to provide a protective environment with fewer inflammatory mediators and reactive oxygen species from the host cord [5]. NSPC graft transplantation without scaffold into the acute SCI lesion exhibited limited survival [5]. Consistent with this observation, only 11.2% of NSPCs survived in animals without AHs whereas survival increased to 38.3% in the AHs-NSPC group 8 weeks post-transplantation (Fig. 6A). Increased NSPCs survival by AHs is certainly one important factor contributing to a higher number of graft-derived axons detected in the host parenchyma (Fig. 8). However, even in the presence of AHs, around 40% of grafted NSPCs were lost in the first 2 weeks post-transplantation. This might be due to the leakage of toxic blood products and acute inflammatory cytokines [41], which impair the survival of grafted NSPCs [42, 43]. Inhibition of inflammatory responses in the acute stage by administering anti-inflammatory drugs [44, 45] and delayed transplantation at a stage when inflammatory responses have subsided [40, 46] might enhance survival of NSPCs and further improve functional outcomes.

## Conclusions

AH scaffolds with anisotropic capillaries cross-linked by Ca^2+^ in combination with NSPCs promote NSPC survival, allow for filling of a spinal lesion cavity, enhance neuronal differentiation of grafted cells and support short axonal regeneration in a linear orientation. NSPC-derived neurons can form reciprocal synapses with host spinal neurons, which might underlie improved electrophysiological conductivity and locomotor outcomes. These promising data support the further investigation of NSPC transplantation in combination with 3-dimensional organized scaffolds to support the formation of neuronal relays across a lesion in the injured spinal cord.

## Supplementary data

Supplementary data are available at *REGBIO* online.

## Funding

This work was supported by the National Natural Science Fund of China (grant number 81901895 to S.L. and grant number 81571242 to Y.W.).

*Conflicts of interest statement*. None declared.

## Supplementary Material

rbac057_Supplementary_Data
