# Supplementary material for: Alginate hydrogel cross-linked by Ca2+ to promote spinal cord neural stem/progenitor cell differentiation and functional recovery after a spinal cord injuryhh
Source: Regen Biomater. 2022 Aug 18;9:rbac057. doi: 10.1093/rb/rbac057 (PMC9438746; doi:10.1093/rb/rbac057)
Supplement: rbac057_Supplementary_Data [file rbac057_supplementary_data.pdf]

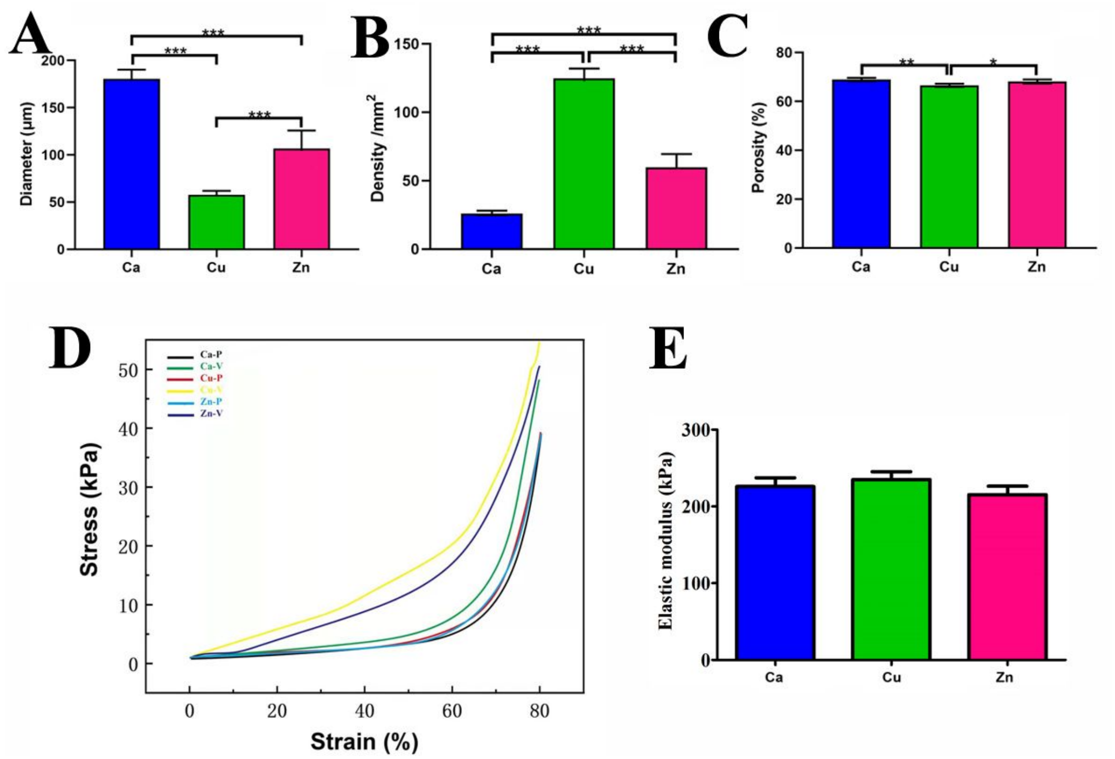

Supplemental Figure 1: Comparison of physical characteristics of AHs cross-linked withCa^{2+}, Cu^{2+}andZn^{2+}, respectively, fabricated under the same conditions. (A) Channel diameter of AHs cross-linked withCa^{2+}, Cu^{2+}, and Zn^{2+} indicates that Ca scaffolds have the largest diameter. (B) Calculation of the number of capillaries per mm^2 shows the highest density in Cu scaffolds. (C) Both Ca and Zn scaffolds have slightly higher porosity than scaffolds cross-linked by Cu^{2+}. (D) Stress-strain curves of Ca, Cu, and Zn scaffolds tested parallel (P) or vertical (V) to longitudinally aligned channels. (E) Elastic modulus of AH scaffolds (*$p< 0.05$; **$p < 0.01$; ***$p< 0.001$; one-way ANOVA followed by Tukey's post-hoc analysis).

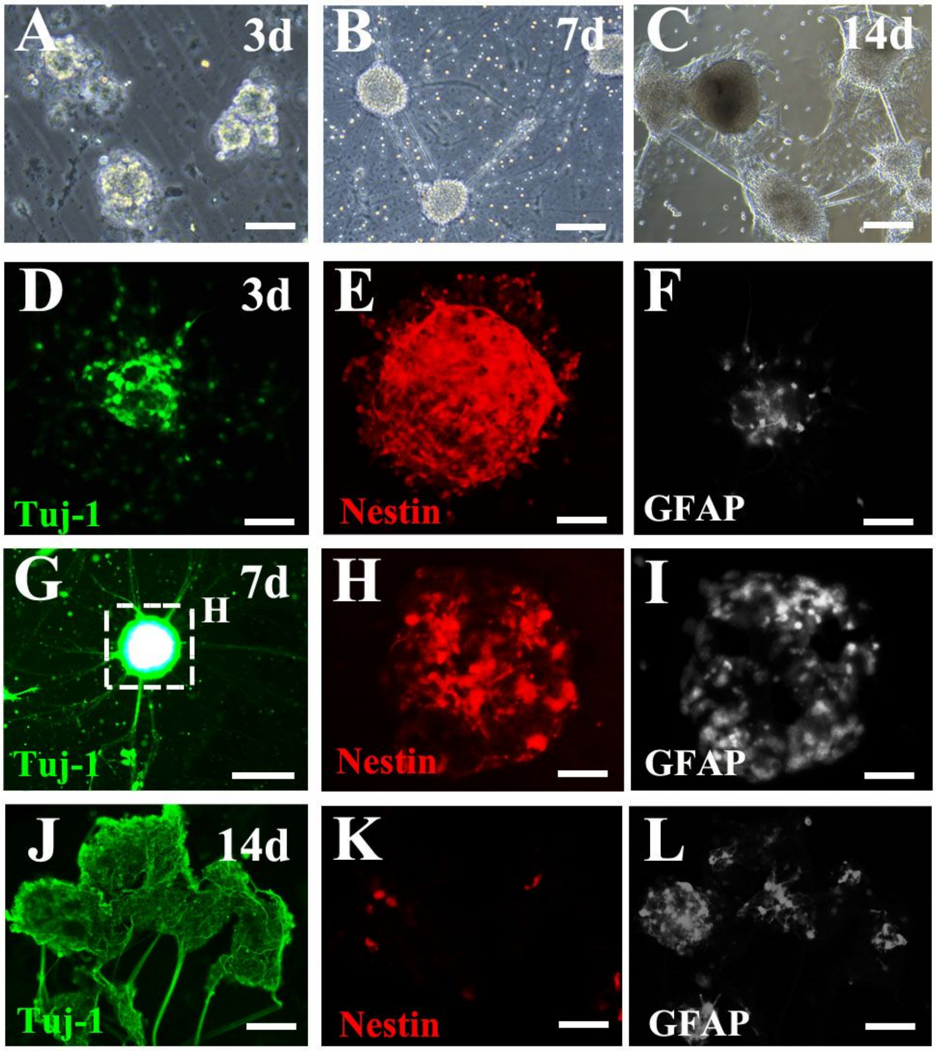

Supplemental Figure 2: *In vitro* culture and differentiation of neural stem/progenitor cells (NSPCs). (A-C) Representative images of E14 rat spinal cord NSPC neurospheres cultured for 3, 7, and 14 days, respectively. (D) Only few cells express Tuj-1 (green), >98% of cells in neurospheres express (E) nestin (red) and a small number of cells express (F) glial fibrillary acidic protein (GFAP) (white)after3days in culture. (G) Outgrowth of neurite bundles immunolabeled for Tuj-1 (green) from neurospheres after cultivation for 7 days. (H–I) Higher magnifications of the area boxed in (G). A proportion of cells express nestin (red) or GFAP (white) in the neurospheres. (J–L) Immunolabeling of adherent neurospheres for (J) Tuj-1 (green), (K) nestin (red), and (L) GFAP. Scale bar: A–C, G, and J–L:

400µm; D–F: 100µm; and H–I: 100µm.

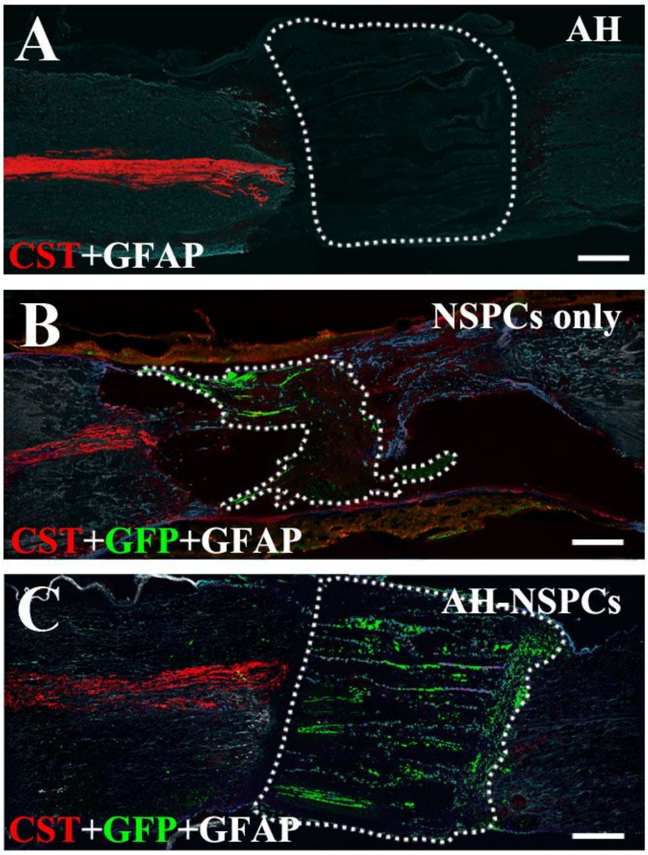

Supplemental Figure 3: Tracing of corticospinal tract (CST) axons in the spinal cord 8 weeks post-injury and -grafting. (A-C) CST tracing in animals grafted with (A) alginate hydrogel scaffolds (AHs), (B) neural stem/ progenitor cells (NSPCs) (green) and (C) AHs pre-seeded with NSPCs. CST axons terminate in the rostral spinal cord labeled for glial fibrillary acidic protein (GFAP) in all groups rather than penetrating the interface into the grafts. Dashed lines indicate the margins of the grafts. Scale bar: A-C, 500µm.
